# A Granular Ontology Model for Maternal and Child Health Information System

**DOI:** 10.1155/2017/9519321

**Published:** 2017-05-16

**Authors:** Saadia Ismail, Majed Alshmari, Khalid Latif, Hafiz Farooq Ahmad

**Affiliations:** ^1^National University of Sciences and Technology (NUST), Islamabad, Pakistan; ^2^College of Computer Sciences and Information Technology (CCSIT), King Faisal University, Alahssa 31982, Saudi Arabia; ^3^COMSATS Institute of Information Technology, Islamabad, Pakistan

## Abstract

In several developing countries, maternal and child health indicators trail behind the international targets set by the UN as Millennium or Sustainable Development Goals. One of the reasons is poor and nonstandardized maternal health record keeping that affects data quality. Effective decision making to improve public healthcare depends essentially on the availability of reliable data. Therefore, the aim of this research is the design and development of the standard compliant data access model for maintaining maternal and child health data to enable the effective exchange of healthcare data. The proposed model is very granular and comprehensive in contrast with existing systems. To evaluate the effectiveness of the model, a web application was implemented and was reviewed by healthcare providers and expectant mothers. User feedback highlights the usefulness of the proposed approach as compared to traditional record-keeping techniques. It is anticipated that the proposed model will lay a foundation for a comprehensive maternal and child healthcare information system. This shall enable trend analysis for policy making to help accelerate the efforts for meeting global maternal and child health targets.

## 1. Introduction

The United Nations Millennium Development Goals (MDGs) numbers 4 and 5 aimed to reduce child mortality by two-third and maternal mortality by three-quarters, respectively, till 2015 [1]. However, several developing regions, including Pakistan, were unable to meet the international targets [2, 3] and the efforts to achieve MDGs were marked as insufficient [4]. For instance, by the year 2015, Pakistan's under 5 mortality rate was 81.1 and the maternal mortality ratio per 100,000 live births was 178 [5]. Situation is not very different in many other developing countries such as Saudi Arabia [6]. The need for improvement resulted as a new set of Sustainable Development Goals (SDGs), which also define targets to deal with global health issues including a reduction in maternal, neonatal, and child mortality in the next 15 years [7].

Achieving maternal and child health (MCH) targets in underdeveloped or developing parts of the world requires significant investment in the infrastructure, improvement in the service delivery, and quality of care [8], as well as the availability of reliable health data [9, 10]. In Pakistan, however, immature e-health solutions are deployed in a limited number of healthcare facilities [2]. The existing nonstandardized record-keeping techniques result in missing records, inconsistencies, poor data quality, and inaccuracies and hence undermine evidence-based decision making in healthcare service delivery [11, 12]. Most of the local hospitals and clinics only have a primitive patient registration systems to record billing information, and electronic records of clinical and medical data are ignored for the most part. In the absence of reliable data, country-level statistics are based on estimates from a selected sample.

Higher level ranking indicators in MCH domain are very well established [13]. Developed countries use more granular data models. Japan, for instance, initiated the Maternal and Child Health Handbook [14] in 1942 (referred as MCHHJ in this manuscript), to create awareness and log necessary information related to pregnancy and delivery, child development, and health education. This handbook greatly contributed in decreasing maternal mortality rate (MMR) and infant mortality rate (IMR) in Japan [15]. Later on, customized handbooks were implemented in many countries such as Bangladesh [16], Indonesia [17], Thailand [18], Cambodia [19], and Mongolia [20]. Our model is also an extension of MCHHJ. However, MCHHJ is a record book so it does not offer some of the features of an information system such as patient scheduling, lab test report, lab orders by a practitioner, and details of procedures and medical examination carried out for patient's assessment.

Fast Healthcare Interoperability Resources (FHIR) is the latest and emerging standard from Health Level Seven International (HL7) for exchanging healthcare data [21]. FHIR exposes various information units, called resources, as granular constituents of medical records. The FHIR specification describes a set of base resources only that may be used in a generic contexts in healthcare. Implementing FHIR in MCH domain, however, requires additional structure definitions and rules about which resource elements and terminologies map to particular MCH requirements. Therefore, the objective of our research is to design and develop a granular data model using existing standards such as HL7 FHIR, MCH Handbook of Japan, and clinical terminologies such as SNOMED for improved record keeping and interoperability in MCH domain (This research is an extended version of our work published in BIBE 2016 [22]).

A web application is also developed to evaluate the usefulness of the proposed model. The system is made accessible to healthcare providers including gynecologist, obstetrician, and pediatrician at partner healthcare facilities. It can be used by expecting women to view their records, and it can also be used by parents to view and maintain health records of neonatal. Feedback was gathered from users. The proposed approach is perceived as more useful compared to traditional record-keeping techniques.

## 2. Methods

The design approach for the data model follows METHONTOLOGY, a methodology for knowledge engineering [23], in an incremental strategy with steps being repeated when necessary. The steps involved in the approach are described as follows:

### 2.1. Requirement Gathering

Specification of the high-level requirements for the model, for example, necessary concepts required to be covered to build a comprehensive and meaningful semantic model were articulated. A set of requirements from two internationally recognized resources, the MCH Handbook in Japan [14] and Common Requirements for Maternal Health Information Systems released by Program for Appropriate Technology in Health (PATH) [24], formed the baseline for the proposed ontology model. An extensive literature review was conducted to gather additional requirements. A list of papers reviewed to collect requirements about which data element is necessary in the MCH domain is given in Table 1.

End users of the ontology model, for example, hospitals, practitioners, patients, researchers, national and international agencies, and government bodies, were defined. Opinion was solicited from local practitioners including obstetricians, gynecologists, pediatricians, and lady health workers (LHW). A list of partner hospitals is mentioned in Table 2. All practitioners consulted during requirement gathering had 5 to 20 years experience of working in a public or private hospital and thus had a deep understanding of patient profiling and hospital procedures. Requirements were translated into use cases as shown in Table 3. For details of these use cases, readers are referred to an online technical document (https://github.com/klatifch/mch).

### 2.2. Semantic Structuring

**Figure 1:**
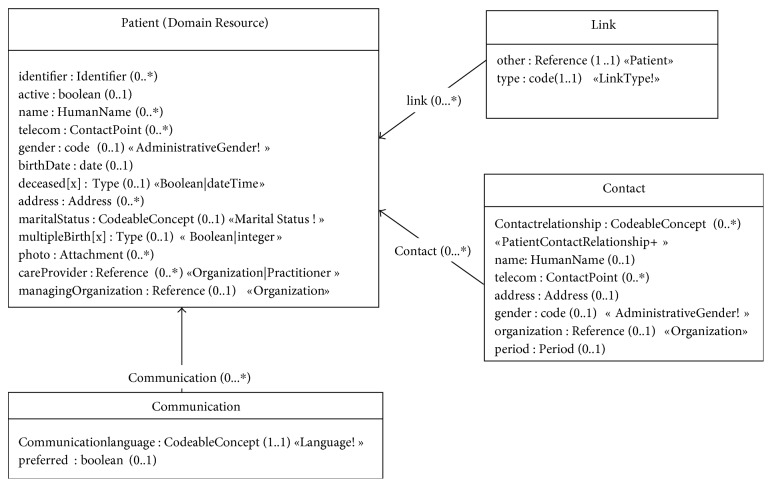
Patient resource model from HL7 FHIR.

A glossary of key domain concepts identified during the requirements specification steps was formalized, and related terms were categorized. Some of the concepts include *Patient* (e.g. *expectant woman*), *Vital sign*, *Symptom*, *Procedure*, and *Allergy intolerance*. Glossary was further extended by adding attributes of the concepts, identifying data types, and modeling relationships among concepts. Key concepts and their attributes were then mapped to HL7 FHIR resources. Some of the concepts had direct mapping, such as *Patient* information is modeled as *Patient* resource (Figure 1 depicts the *Patient* resource as in HL7 FHIR). Some of the concepts were mapped to their equivalent resources having a different name, such as *Observation* resource from HL7 is used for recording vitals. See Table 4 for details of these mappings. It is worth pointing out that many FHIR resources heavily benefit from concepts in other code systems such as SNOMED and LOINC. For instance, AllergyIntolerance resource may use the following concept from SNOMED:

 
"identifier":[{
 
"label": "House dust allergy",
 
"system": "http://snomed.info/sct",
 
"value": "232349006" }]

Model validation was performed by the selected practitioners, and their feedback was used to correct inconsistencies. Suitability of the model was evaluated in combination with an application infrastructure as explained in the later section.

### 2.3. Mapping with Indicators

The commission on information and accountability for women's and children's health has selected 11 core indicators on maternal, child, and neonatal health, aligned with indicators of MDGs [29]. Amongst these, our model provides estimation of the impact indicators: maternal mortality ratio and under five child mortality. It can also help determine the outcome indicators such as medications provided to patients for treatment of illness.  Table 5 details the mapping of the model to MCH indicators. A list of key healthcare indicators was formed such as maternal and neonatal mortality and morbidity, socioeconomic and demographic determinants of health, and behaviour during pregnancy related to exercise, diet, and nutrition intake. These indicators were also mapped to HL7 FHIR resources in our data model.

## 3. The Proposed System

The design and development of proposed MCHR system involved the following major steps:

1. Designing MCH system architecture
2. Designing underlying database structure
3. Implementation of the MCH system using proposed data model.

### 3.1. MCH System Architecture

**Figure 2:**
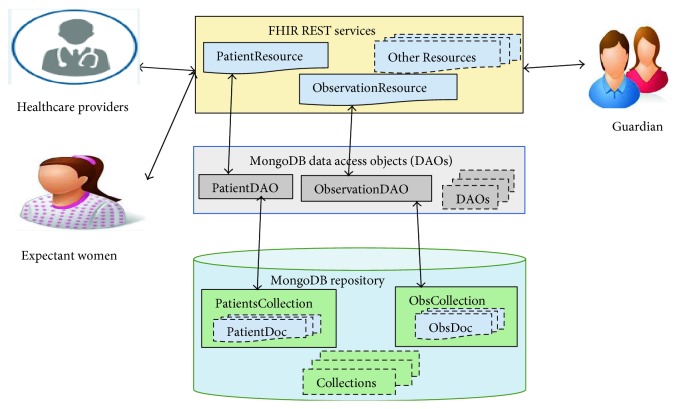
Proposed maternal and child health record system architecture.

A modular architecture for the proposed MCH system is explained in this section.  Figure 2 depicts the choice of technologies in different modules and layers. The modules involved in the architecture are described in detail as follows:

### 3.2. Database Structure

FHIR resources may be serialized either as XML or as JSON documents. JSON format being lightweight is preferred in modern web applications for data exchange. The mother and child data objects are mapped to FHIR resources in the proposed system and are processed as JSON objects. Hence, MongoDB as a native JSON storage system [30] was used. It stores data as JSON-formatted documents. Moreover, MongoDB scales well as compared to relational databases for document models. This is because join-based queries are not required as all the relevant data of an entity is present in a single-JSON document. This flexibility can help in overall system scalability.

**Figure 3:**
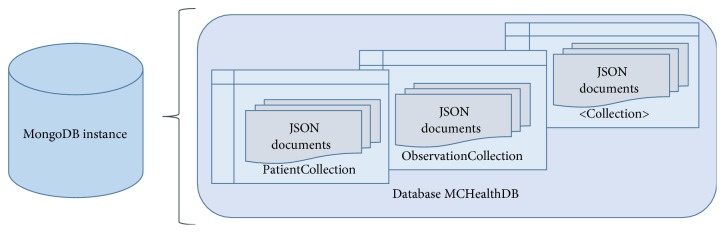
MongoDB physical data model.

Though MongoDB is schema-free but, an underlying structure (schema) was defined in order to ensure conformance with the proposed data model. Each collection in the database represents a category of resources, such as “Patient,” “Practitioner,” and “Organization.” Each patient-related document, for instance a lab report, is placed as a nested JSON document within the relevant patient resource. This is different from traditional relational databases where items are stored in separate tables and are linked through foreign keys. In contrast, a patient resource in MongoDB represents a comprehensive record of all related actions and outcomes.  Figure 3 represents this data model.

**Figure 4:**
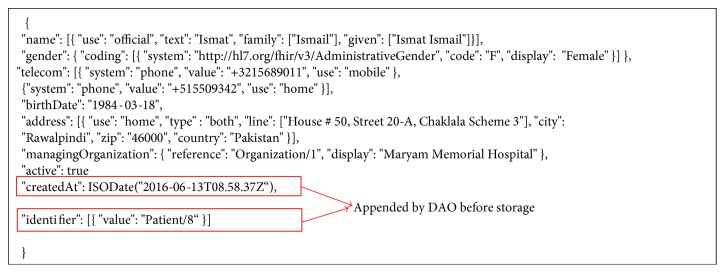
A patient resource as JSON document.

The FHIR documents received from the client-side web applications are parsed into JSON documents by the data access objects (DAOs). Data objects provide an interface to the underlying MongoDB repository, validate the payloads, append identifiers, and transfer contents to the MongoDB repository for storage in an appropriate collection. Data objects are also responsible to return the results of queries performed by the client.  Figure 4 shows an abridged example of a “Patient” resource as MongoDB JSON document.

#### 3.2.1. FHIR-Based RESTful API

This layer consists of the RESTful services that process the requests received from a client. The clients includes both the MCH system and other external systems to enable integration with existing information systems used in the partner hospitals. The REST web services are defined for each resource category corresponding to FHIR specification, to enable the create, read, search, update, and delete operations on these resources. The services respond JSON objects to be consumed at the client side. We have defined the URI templates to which a web service responds.  Table 6 contains some generic service paths and their descriptions that are followed for access.

## 4. Evaluation

To determine the effectiveness of the proposed model, a web-based application was implemented. Five healthcare providers from partner hospitals that were involved in requirement-gathering phase as well as 30 women who were pregnant or had a baby within last 6 months used the system and provided a candid opinion regarding the system's effectiveness.  Table 7 lists the characteristics of mothers interviewed for system evaluation. Participants were required to perform certain tasks. Healthcare providers were asked to register patients on the system and add their personal data and clinical history. The patients were able to view this record. They also checked their prepregnancy and current BMI on providing their weights and heights to know if the BMI was appropriate according to the gestational age. These users later on filled a questionnaire to record their opinion about the system. The questionnaire targeted information about women's or healthcare providers' characteristics.  Table 8 lists the questions asked from participants. It is worth pointing out that six of the participant mothers had no formal education and were trained to use a computer-based system.

The system was evaluated on the basis of user opinion regarding its usability and effectiveness. The answers to the questions asked were categorized depending upon the level of confidence that users showed in this system.  Table 9 presents a summary of this evaluation. Each evaluation criteria is further explained in subsequent sections.

### 4.1. Understanding System Features

The healthcare providers and majority of women understood all the functionalities that the system offers. However, some women from weaker educational background could not comprehend even very simple features such as BMI value and range.

### 4.2. Approval of Effectiveness

Several users found the system as very effective in improving the current record maintenance techniques, contributing towards a better and efficient healthcare delivery system. The majority of healthcare providers stated that manual procedures overburden the doctors as they have to see the patients and create the records twice—once for the patients, that is, prescriptions and then for hospital registers. This process is inefficient, time consuming and prone to errors. The maintenance of hospital registers is hard causing space issues and making it cumbersome to locate patient files for future references. Patients face risks of losing their manual records. This is critical especially in pregnancy-related data because the records, once lost of earlier time span (trimester), cannot be obtained in a later stage of pregnancy. The unavailability of a systematic means of recording previous histories results in patients being asked the same questions on each encounter regarding their pregnancy/medical history. Similarly, the diagnostic tests or medications, that need only be used once or for a particular time span, may also be repeatedly prescribed.

However, some found it unsuitable, especially for women belonging to underprivileged areas that lack resources. Similarly, the lack of education and awareness was also identified as one of the big reasons for not being able to use the system. One of the LHWs stated, “Women in the villages usually do not disclose their pregnancies unless it becomes very obvious in later stages. They are also not willing to have monthly check-ups, ultrasounds or medications even if provided free-of-cost by the government. LHWs also require training and education for being able to use these systems.”

### 4.3. Level of Usefulness

Most of the patients, especially those doing jobs, acknowledged the system features as very practical and useful in helping them track health status. However, some of them, due to lack of resources, education, or awareness, did not think that they could use the system on their own. However, they acknowledged the benefits of viewing health record on an online health system and the ability to track health status by recording weight or calculating BMI and getting suggestions online from the physician.

### 4.4. Willingness to Use in Future

Several doctors and women agreed to use the system considering the level of ease it provides as compared to traditional record keeping. However, some of them felt more comfortable in keeping the file-based records. Although they acknowledged the risks of losing their manual records and also have had the experience of misplacing their files, they still did not feel the desire/need to change. A woman stated, “I lost my previous records but it is okay. I shall see the doctor, get the scans done again and thus have my new records.”

Some of the women, however, were incapable of using the system because of lack of education or resources. There were also some, who were not interested in using the system. Some social issues were identified as reasons for their unwillingness. One woman said, “I would prefer leaving the household and taking a break, be it a doctor's visit.” They also felt that their husband, mother-in-law, or other family members will not trust their opinions on the basis of health information they interpret from an online health record system.

### 4.5. Evaluation Summary

The system provides all the relevant functionalities starting from patient registration to patient-doctor encounter details to a prescription for the patient and record maintenance for later reference. The proposed system also provides access to patients for viewing their medical records. Moreover, the capability of adding blood pressure and weight by the patient for review by the health care provider was also added. However, doctors' opinion reflected that measurements entered by patients are not trustworthy. So, for the time being, an ability to add only height and weight is provided to the patient. The patients can also view their body mass index (BMI). The system automatically generates a message indicating whether the calculated BMI is appropriate or not according to the stage of pregnancy. This is helpful in identifying the health and appropriate weight gain of a patient and for taking suitable measures for improving the diet and habits of a patient for a healthy pregnancy. According to some of the doctors, carrying out researches on existing data sources in Pakistan is challenging because of the poor data collection efforts. However, the proposed approach can provide very granular data to the doctors or researchers to facilitate analysis and policy making. The system also enables maintenance of a child's health record and allows parents/guardian to view the record of their child from the beginning.  Table 10 describes the benefits of the proposed system over the existing record-keeping options. At the same time, there were various limitations of the system as well. Some of these limitations or challenges involved in implementation include the following:

1. National HIT policies are difficult to enforce in an effective and secure environment in the absence of government support.
2. People are generally less motivated and apprehensive to adapt to new technologies because of cultural and social reasons.
3. Lack of adequate technological infrastructure (hardware, communication channels, and internet) is a huge barrier in a widespread implementation. People in rural areas who do not have Internet and other technical facilities available were not able to use it.

## 5. Conclusion

Though a preliminary implementation of the proposed system was carried out, the evaluation study suggests that the MCH system can enable reliable and efficient record keeping. It can be a catalyst in the availability of quality data to facilitate analysis, research, and evidence-based decision making. However, additional work is needed to rollout a comprehensive MCH information system at a wider scale to facilitate the manipulation, analysis, and dissemination of health data pertaining to a mother as well as child health to help achieve the global health targets at the national level.

The biggest challenge identified in system rollout was the lack of education and awareness. The adult female literacy rate for Pakistan is only 45% [31]. Therefore, we conclude that a significant effort is required in training and educating people for the use of a comprehensive health record system. Moreover, healthcare providers generally feel intimidated in using a computerized system compared with the traditional approaches.

## Figures and Tables

**Table 1:** Sources for requirement gathering.

Reference	Concepts of MCHIS
[24]	(i) Scheduling
	(ii) Diagnostics including laboratory tests
	(iii) Medication management

[25, 26]	(i) *Outputs*: in the form of information that can form the basis for evidence-based decision making. This highlights the importance of patient's *diagnostic/lab reports*
	(ii) *Indicators*: such as socioeconomic, environmental, behavioral, and demographic determinants of health; data on maternal attitudes and experiences before, during, and shortly after pregnancy; and also, the indicators that determine health status such as rates of mortality and morbidity

[27, 28]	(i) *Information access*: users are able to view maternity information through a website.

**Table 2:** Partner hospitals and clinics for requirement gathering.

Title	Location	Beds
Military Hospital (MH)	Rawalpindi	1200
Combined Military Hospital (CMH)	Rawalpindi	1000
Medicsi Clinic	Islamabad	50
Shifa International Hospital	Islamabad	500
Anwar Clinic	Rawalpindi	100
Nusrat Clinic	Rawalpindi	50

**Table 3:** High-level use cases for the data model.

Sr.	Use case	Frequency
1	Manage patient registration	Once
2	Manage patient history	Once
3	Schedule encounters	Multiple
4	Manage vitals	Multiple
5	Manage pregnancy profile	Multiple
6	Maintain record of treatment	Multiple
7	Maintain record of outcome	Multiple
8	Manage labor and delivery plan	Once
9	Labor and delivery record	Once
10	Manage neonatal health record	Multiple

**Table 4:** Data entities and mappings with HL7 FHIR resources.

Data entities	FHIR resources
*(1) Patient registration*	
Personal information, emergency contact	Patient
Marriage details, previous children	RelatedPerson

*(2) Patient history*	
Illness, infections, pregnancy history	Condition
Assertions related to illness	Observation
Surgical history	Procedure
Medication history	MedicationPrescription
Allergy history	AllergyIntolerance
Family history	FamilyHistory
Social history (such as tobacco use)	Observation

*(3) Current pregnancy record/encounters*	
Encounter details	Encounter
Health status/characteristics	Observation
Weight and height for BMI calculation	Observation
Baby's characteristics	Observation
Lab reports, diagnostic tests	DiagnosticReport

*(4) Record of treatment*	
Suggesting tests/scans	DiagnosticOrder
Suggesting medications/food supplements/advice	MedicationPrescription
Conduct ultrasounds	Procedure

*(5) Labor and delivery record*	
Plan for method of delivery	CarePlan
Baby's health status	Observation
Birth outcome (e.g., live or still)	Observation

**Table 5:** Indicator mapping.

Indicator type	Indicators/examples	Concept of the model	Corresponding FHIR resource
Impact	Maternal mortality ratio	Record of mother's death	Observation
	Under five mortality	Record of neonatal death/still birth	Observation
Outcome	Proportion of women treated for an illness	Patient	Patient
		Medication	MedicationPrescription
		Surgery	Procedure
		Illness	Condition

**Table 6:** REST resource URIs.

URL pattern	Service description
POST/resource	Enables creation of resource in particular collection. Such as a “Patient”
GET/resource	Lists resources of a particular type such as a list of all patients
GET (or POST)/resource/{id}	View (or edit) a particular resource specified by the identifier
GET/resource/_search?[criteria]	List resources of particular type meeting the given search criteria
GET/patient/{id}/bmi	Analyze if BMI is appropriate according to prepregnancy BMI and current gestational age

**Table 7:** Characteristics of participants (mothers).

Characteristics	Number	Percentage
*Age*		
18–24	3	10
25–34	21	70
36–45	6	20
*Religion*		
Muslims	27	90
Non-Muslims	3	10
*Formal school education*		
No	6	20
Yes	24	80
*Employment status*		
No	21	70
Yes	9	30
*Pregnancy status*		
Pregnant	12	40
Gave birth within last six months	18	60
*Gravida*		
Primi	12	40
Multi	18	60

**Table 8:** List of questions asked from participants.

Questions from mothers	Questions from healthcare providers
Were they already seeing a doctor or visiting a healthcare facility?	For how long have they been working?
Have they ever used a digital system before?	At which healthcare facilities have they worked?
Do they understand the purpose of the system and its features?	Current modes of recording health data that they use?
Do they find it useful?	Have they ever used a digital EMR before?
Comments for improvement	Computer usage skills?
	Opinion regarding system's acceptability?
	Comments for improvement?

**Table 9:** Opinions of test users about MCHRS.

Question	Category	Count	Percentage
Extent of understanding	All features	32	91.4
	Only common features	3	8.6
Level of effectiveness	Very effective	29	82.86
	Unsuitable (considering issues of resources)	2	5.71
	Unsuitable (lack of education/awareness)	4	11.43
Level of usefulness (patient's perspective)	Very useful	31	88.6
	Unsuitable (considering issues of resources)	2	5.7
	Unsuitable (lack of education/awareness)	2	5.7
Level of willingness to use the system	Definitely	26	74.3
	Reluctant (comfortable in manual records)	3	8.6
	Reluctant (incapable of using)	4	11.4
	Not interested	2	5.7

**Table 10:** Comparison of proposed MCH system with existing techniques.

Feature	Proposed	Techniques in use	OpenEMR	OpenMRS
HL7 FHIR or other health standard for information modeling	Yes	No	Standard-based medical billing	HL7 engine for data import
RESTful web services	Yes	No	No	Yes
MongoDB or other NoSQL data stores	Yes	No	No	No
Open-source web-based solution	Yes	No	Yes	Yes
Access for patients	Capability to view all personal records	Some provide access to lab reports only	Yes	Yes
	Calculation of BMI			
Record of complete patient history (past pregnancies, medications, allergies, surgeries)	Yes	Few hospitals maintain discharge summaries of inpatients and lab reports	Yes	Yes
Minimal chances of losing patient records	Yes	No	Yes	Yes
Easier record maintenance	Yes	No	Yes	Yes
Efficient retrieval	Yes	No	Yes	Yes
Reliable data for research and analysis	Yes	No	Yes	Yes
